# Developing and Implementing a Web-Based Relapse Prevention Psychotherapy Program for Patients With Alcohol Use Disorder: Protocol for a Randomized Controlled Trial

**DOI:** 10.2196/44694

**Published:** 2023-01-25

**Authors:** Jazmin Eadie, Gilmar Gutierrez, Elnaz Moghimi, Callum Stephenson, Payam Khalafi, Niloofar Nikjoo, Jasleen Jagayat, Tessa Gizzarelli, Taras Reshetukha, Mohsen Omrani, Megan Yang, Nazanin Alavi

**Affiliations:** 1 Department of Psychiatry Queen's University Kingston, ON Canada; 2 Department of Psychology Queen’s University Kingston, ON Canada; 3 Department of Education Queen’s University Kingston, ON Canada; 4 Centre for Neuroscience Studies Queen’s University Kingston, ON Canada; 5 OPTT Inc Toronto, ON Canada

**Keywords:** mental health, alcohol use disorder, psychotherapy, eHealth, cognitive behavioral therapy, online, internet, treatment, electronic care

## Abstract

**Background:**

Alcohol use disorder (AUD) is characterized by problematic alcohol use accompanied by clinically substantial distress. Patients with AUD frequently experience high relapse rates, and only 1 in 5 remain abstinent 12 months post treatment. Traditional face-to-face relapse prevention therapy (RPT) is a form of cognitive behavioral therapy (CBT) that examines one's situational triggers, maladaptive thought processes, self-efficacy, and motivation. However, access to this treatment is frequently limited due to its high cost, long waitlists, and inaccessibility. A web-based adaptation of RPT (e-RPT) could address these limitations by providing a more cost-effective and accessible delivery method for mental health care in this population.

**Objective:**

This study protocol aims to establish the first academic e-RPT program to address AUD in the general population. The primary objective of this study is to compare the efficacy of e-RPT to face-to-face RPT in decreasing relapse rates. The secondary objective is to assess the effects of e-RPT on quality of life, self-efficacy, resilience, and depressive symptomatology. The tertiary objective is to evaluate the cost-effectiveness of e-RPT compared to face-to-face RPT.

**Methods:**

Adult participants (n=60) with a confirmed diagnosis of AUD will be randomly assigned to receive 10 sessions of e-RPT or face-to-face RPT. e-RPT will consist of 10 predesigned modules and homework with asynchronous, personalized feedback from a therapist. Face-to-face RPT will comprise 10 one-hour face-to-face sessions with a therapist. The predesigned modules and the face-to-face sessions will present the same content and structure. Self-efficacy, resilience, depressive symptomatology, and alcohol consumption will be measured through various questionnaires at baseline, amid treatment, and at the end of treatment.

**Results:**

Participant recruitment is expected to begin in October 2022 through targeted advertisements and physician referrals. Completed data collection and analysis are expected to conclude by October 2023. Outcome data will be assessed using linear and binomial regression (for continuous and categorical outcomes, respectively). Qualitative data will be analyzed using thematic analysis methods.

**Conclusions:**

This study will be the first to examine the effectiveness of e-RPT compared to face-to-face RPT. It is posited that web-based care can present benefits in terms of accessibility and affordability compared to traditional face-to-face psychotherapy.

**Trial Registration:**

ClinicalTrials.gov NCT05579210; https://clinicaltrials.gov/ct2/show/NCT05579210

**International Registered Report Identifier (IRRID):**

PRR1-10.2196/44694

## Introduction

Alcohol use disorder (AUD) is a mental health disorder that affects approximately 3.6% of the global population aged 15 to 64 years [1]. The disorder is highly prevalent and a major contributor to illness and mortality worldwide [2,3]. AUD is characterized by impaired control over alcohol use, leading to physiological dependence and negative psychological, social, and physical consequences [3,4]. The disorder is associated with many physical and psychiatric comorbidities, such as major depressive disorder, generalized anxiety disorder, liver cancer, and hypertension [2,5-7].

The rise in mental health challenges during the COVID-19 pandemic resulted in growing concerns over the health behaviors of individuals with AUD [8]. Stay-at-home orders presented complex relationships between financial issues, low social interaction, and anxiety about the future [9]. Moreover, the disruption to clinical services is believed to have contributed to increased relapse and subsequent negative health outcomes for people with AUD [10]. Kim et al [11] demonstrated that out of 182 participants with previous AUD, 23% increased their consumption during the lockdown. Additionally, 17% of the subjects abstained for an average of 19.5 months before relapsing during lockdown [11].

AUD treatments typically involve abstinence from alcohol due to the high failure rates and risks associated with controlled drinking approaches [12,13]. However, AUD is known as a “relapsing condition,” since patients often relapse following abstinence [14]. Within the AUD framework, a lapse is defined as a single episode of drinking, whereas a relapse involves a return to a pattern of problematic drinking [15]. Stillman et al [16] found that relapse rates in people with AUD are approximately 60% to 80% after 3 months of treatment and 70% to 80% after 12 months post treatment. These rates highlight the importance of integrating relapse prevention into AUD treatment programs. These programs identify personal factors that contribute to relapse and offer helpful strategies to improve coping with cravings, and mood fluctuations, and identify triggers to decrease the rate and severity of relapses [17].

Psychosocial interventions are commonly used to treat AUD, with cognitive behavioral therapy (CBT) being one of the most studied psychological approaches [18,19]. CBT for AUD encourages abstinence in the presence of triggers and cravings by targeting maladaptive thought processes and behaviors [20,21]. This therapeutic approach has demonstrated greater efficacy compared to no treatment or minimal treatment [22]. Relapse prevention therapy (RPT) is a validated CBT treatment for AUD that targets both interpersonal and intrapersonal factors such as motivation and trigger identification. RPT also equips patients with coping strategies to use in situations where alcohol may be available [23-25]. The efficacy of RPT can vary depending on the substance used, personal factors such as income, and comorbid disorders [26,27]. However, despite the effectiveness of this type of therapy, financial issues, accessibility problems, and logistical problems may make it challenging for individuals to receive face-to-face care [28]. Moreover, individuals with AUD frequently experience shame and stigma surrounding their diagnosis, which can reduce treatment seeking in this population [29].

To address some of the issues of accessibility and availability, web-based adaptations of traditional face-to-face psychotherapy, such as internet-based CBT (e-CBT) have flourished in recent years. Web-based psychotherapy offers several advantages, such as not requiring office space, placing lower demands on therapists’ time, improving accessibility, etc [30]. Although the literature has shown comparable effectiveness between web-based psychotherapy and face-to-face psychotherapy in the management of AUD [31], and face-to-face RPT has demonstrated beneficial effects in relapse prevention [12,24], web-based RPT (e-RPT) remains an understudied option for the management of AUD [32].

Therefore, this study proposes the development, study, and implementation of an e-RPT program for the management of AUD. The proposed program could be a viable solution to address the potential accessibility, stigma, and financial issues presented by traditional in-person therapies [32]. Thus, the primary objective of this study is to compare the efficacy of a secure e-RPT program to face-to-face RPT to decrease relapse rates in patients with AUD. Based on the evidence comparing face-to-face and web-based psychotherapies in other disorders, we hypothesize that both face-to-face RPT and e-RPT will have similar effectiveness in AUD symptom management, reducing alcohol consumption, and lowering relapse rates [33,34]. The secondary objective is to assess the effects of online treatment on quality of life, self-efficacy, resilience, and depressive symptomatology. The tertiary objective is to evaluate cost-effectiveness by comparing the amount of time an e-RPT therapist spends with each patient to the therapists administering face-to-face RPT. The results of this study will supplement the available literature and aid in the development of clinical recommendations for the management of AUD.

## Methods

### Study Design and Participants

This is the protocol for a randomized controlled trial studying the efficacy of a novel e-RPT program. Participants (n=60) diagnosed with AUD will be recruited through physician referrals from Kingston Health Sciences Centre, family physicians, other health care providers, and self-referrals within Kingston, Ontario. After providing consent to participate in the study, a Mini International Neuropsychiatric Interview 7.0.2 (MINI) will be conducted to confirm the diagnosis of mild to moderate AUD [35]. The MINI is a diagnostic interview that assesses 17 common mental disorders by following the diagnostic criteria of the *Diagnostic and Statistical Manual of Mental Disorders*, Fifth edition [35]. After the MINI, the Readiness to Change Questionnaire (RCQ) will be administered in an interview format to investigate the individual's desire to change their alcohol consumption [33]. In addition to the RCQ, participants will be asked questions listed in Textbox 1 to capture their history with AUD. Answers to these questions, along with the intake MINI, will be reviewed with the principal investigator (PI) to ensure that the participant meets eligibility criteria and to confirm the AUD diagnosis.

**The 6 additional interview questions to establish eligibility.**

1. Have you been diagnosed with alcohol use disorder (AUD)?
2. If applicable, when were you diagnosed with AUD?
3. Are you currently enrolled in another relapse prevention program?
4. In the past month, how many times have you had an alcoholic beverage?
5. If applicable, how many drinks did you have on average in those times?
6. If applicable, how many drinks did you have per week on average in the past month?

Participant inclusion criteria are being 18 years or older at the start of the study and having a diagnosis of mild to moderate AUD according to the MINI (2 to 5 MINI AUD symptom criteria) [20], competence to consent and participation, ability to speak and read English, and consistent and reliable access to the internet. Additionally, contemplation about stopping or having stopped consuming alcohol in the past 30 days is required. This will be assessed by the RCQ (participants should be in the contemplation or action stage according to the questionnaire) and by asking them about when they stopped drinking or began considering alcohol cessation [34,35]. Exclusion criteria are acute hypomanic or manic episodes, acute psychosis, other active substance use disorders classified as moderate or severe, active suicidal or homicidal ideation, untreated clinically substantial somatic symptoms or mental disorders, or current enrollment in another relapse prevention program. Additionally, men who consumed more than 4 drinks per day or 14 drinks per week and women who drank 3 drinks per day or 7 drinks per week in the past month will be excluded [36].

### Procedure

**Figure 1:**
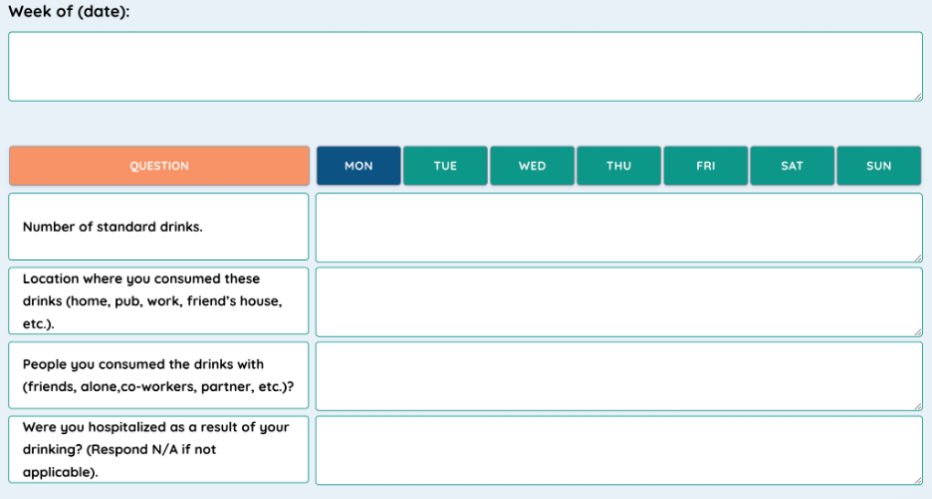
Daily drinking diary.

Upon completion of the eligibility assessments, participants will be randomly assigned through a computerized system to one of the 2 groups: e-RPT or face-to-face RPT. Participants will be equally stratified (e-RPT n=30; face-to-face RPT n=30). These treatments will be delivered as an augmentation to treatment as usual (eg, medications, regular physician or clinician visits, referrals, or consultations that are conducted outside of this research study). The e-RPT program will be delivered through the Online Psychotherapy Tool (OPTT), a secure and interactive platform developed by the PI [37]. The face-to-face RPT program will be delivered through videoconference sessions using Microsoft Teams. Participants in the e-RPT and face-to-face groups will also have access to OPTT to access the triage module, the daily drinking diary (Figure 1), and assessment questionnaires.

### Triage Module

The triage module is a short module intended to introduce clients to the RPT program and the drinking diary. All participants in the e-RPT and face-to-face groups will complete this module before starting their respective treatments.

### e-RPT

Participants in the e-RPT group will receive 10 predesigned online modules per week on the OPTT platform. Each module consists of approximately 30 slides and should have an estimated completion time of 45 minutes. At the end of each module, participants will be assigned homework to be completed before their next module is assigned. The therapists will submit personalized feedback by using session-specific therapy feedback templates. These structured templates standardize the quality of feedback across different patients and therapists. In previous studies, therapists have been able to effectively use these templates to prepare feedback in approximately 15-20 minutes [30].

This e-RPT program will focus on teaching essential cognitive and behavioral skills such as identifying maladaptive thought processes, increasing engagement in day-to-day activities, and developing strategies to reduce alcohol consumption. The content of the sessions is outlined in Table 1.

**Table 1:** The program outline and session summary for the 10-week web-based and face-to-face relapse prevention program.

Session number	Session subject
1: Understanding addiction	Introduces relapse prevention and what to expect from the course. The session explains what addiction is, including the symptoms, signs, consequences, and different types of addiction. Participants will create a SMART goal that they will try to achieve throughout the program. They will also share their personal experiences with addiction.
2: Exploring and strengthening your motivation	Focuses on the 5 stages of motivation and the role of motivation in relapse prevention. Helpful techniques are discussed to help them reflect on and boost motivation throughout their recovery journey. Participants will create a pros and cons list about stopping alcohol use and reflect on different aspects of their ability to stop using alcohol.
3: Triggers	Describes what triggers are and how to recognize them, including both internal and external triggers. This session also provides strategies to handle triggers through avoidance and careful planning. Participants will learn to identify their triggers and to be prepared for them if encountered.
4: Handling cravings and signs of relapse	Presents skills for dealing with cravings as well as identifying and preventing a potential relapse. For instance, applying distraction methods during stressful situations decreases their urge to consume alcohol. Participants will also identify their warning signs by reflecting on previous relapse experiences.
5: The 5-Part Model	The 5-Part Model will be explained, which highlights the importance of differentiating between our thoughts, feelings, physical reactions, and behaviors in stressful situations. Participants will complete the 5-Part Model using an example from their lives.
6: The Thought Record—Part 1	Introduces the thought record and its relation to cognitive restructuration to highlight the connection between feelings, behaviors, and thoughts. This session focuses on the first 3 columns of the Thought Record (out of 7 columns). The first 3 columns include the situation, followed by the feelings and automatic thoughts associated with the situation. Participants are asked to complete the first 3 columns of a thought record for a stressful situation they experienced within the past week.
7: The Thought Record—Part 2	This session continues with the remaining 4 columns of the Thought Record, including gathering evidence in support of and against the identified automatic thoughts and finding alternative and balanced thoughts after examining the evidence. Participants will learn to challenge irrational thoughts using the thought record. They will also learn how to replace these irrational thoughts with more balanced and helpful ones. Participants have to complete a full thought record of a stressful situation related to their AUD within the past week.
8: Thinking errors and the Activity Record	Explores irrational thinking and common thinking errors. This session also presents and highlights the importance of the Activity Record, a tool designed to record and plan weekly activities. Participants are asked to complete an activity record for an entire week.
9: Mindfulness and breathing techniques	Presents mindfulness, breathing techniques, and other helpful skills to reduce urges and cravings associated with alcohol and to promote increased awareness. Participants are asked to incorporate one or more of these practices into their daily routines for a week and to reflect on how these practices made them feel. Participants will also identify which, if any, practices they found to be most effective.
10: Review	This session is a review of the program. It summarizes all of the useful tools and techniques for relapse prevention that were described throughout the program. Participants are asked to reflect on the activities that they found to be the most effective throughout the sessions.

### Face-to-Face RPT

Participants in the face-to-face intervention will meet with their therapist weekly through videoconferencing on Microsoft Teams [38]. During these 1-hour sessions, therapists will follow the same 10-week structure and content as e-RPT (Table 1). In contrast to e-RPT, where homework is written web-based by the therapist, the homework for the face-to-face sessions will be reviewed with the participant during the live session. At the end of each session, participants will receive the same homework and feedback as the e-RPT condition.

### Training

Therapists for both e-RPT and face-to-face RPT will consist of research assistants who are trained in RPT and writing feedback. All therapists are trained by the PI, who is an expert in the electronic delivery of psychotherapy [30,39,40]. During training, the PI will closely guide the therapists through their first patient (assigning modules, reviewing homework, writing feedback, and conducting face-to-face sessions). All therapists will be supervised by the PI to ensure the quality and reliability of the treatment programs. To ensure the quality of the feedback, therapists will practice writing it using structured templates. All face-to-face sessions and feedback will be reviewed with the PI before being sent to the participants.

### Outcome Measures

The primary objective of this study will be to determine if e-RPT has similar effectiveness to face-to-face RPT at reducing alcohol consumption and preventing relapses. This study defines relapse as either the consumption of at least 2.5 oz. of pure ethanol, which is around 4 and a half standard drinks (Canadian guidelines for standard drinks: 12 oz of beer with 5% ethanol, 12 oz of cider with 5% ethanol, 5 oz of wine with 12% ethanol, and 1.5 oz of spirits with 40% ethanol) [41,42] on one occasion or hospitalization because of alcohol drinking [43]. Therefore, to determine the effectiveness of this program, daily alcohol consumption will be recorded in a drinking diary accessible through OPTT. This diary will ask the participants to report how many drinks they consumed on each day of the week, where they had those drinks, with whom, and if their alcohol consumption resulted in hospitalization (Figure 1).

The study will also determine whether e-RPT and face-to-face RPT have a beneficial effect on self-efficacy, quality of life, resiliency, coping behaviors, and depressive symptomatology. These metrics will be assessed through the following scales: Situational Confidence Questionnaire (SCQ-100) [44], 14-item Resilience Scale (RS-14) [45], Coping Behaviors Inventory (CBI) [46], Patient Health Questionnaire (PHQ-9) [47], and Quality of Life Enjoyment and Satisfaction Questionnaire—Short Form (Q-LES-Q-SF) [48]. These assessment scales will be completed at study entry (baseline), amid the program (week 5), and post treatment (week 10). After the treatment period, we will conduct follow-up assessments with all our outcome measures at 3, 6, and 12 months. The amount of time that e-RPT therapists spend writing feedback compared to the 1-hour face-to-face session will be used as a measure of therapists’ time efficiency and will be tracked on the OPTT platform.

### Ethical Considerations

All components of this study were reviewed for ethical compliance by the Queen’s University Health Sciences and Affiliated Teaching Hospitals Research Ethics Board (File #: 6033212) in April 2022. Participants are only identifiable by an ID number, and all records are stored on a secure platform. Participant data is only accessible by the care providers, and anonymized data is provided to the analysis team. Participants can withdraw from the study at any point and request that their data be removed from the analysis. The research team will safeguard the privacy of the participants to the extent permitted by the applicable laws and their duty to report.

OPTT is compliant with the Health Insurance Portability and Accountability Act, Personal Information Protection and Electronic Documents Act, and Service Organization Control 2. All servers and databases are hosted in Amazon Web Service Canada cloud infrastructure to assure provincial and federal privacy and security regulations are met. OPTT does not collect identifiable personal information or IP addresses. OPTT collects anonymized metadata to improve service quality and provide advanced analytics to the clinician team.

### Data Analysis

The data will be initially assessed for outliers, missing, and nonsensical variables. These variables will not be imputed, since they will be handled as missing. Similar studies that involved CBT and e-CBT demonstrated a dropout rate of up to 40% for both conditions once the study concluded [49]. Thus, this study will intentionally oversample the experimental and control groups to account for this dropout rate. Considering the effect size for face-to-face RPT reported by Harada et al [50] (2021) for CBI (effect size=0.65), a significance level of α=.05 and a power of 0.90, a sample size of 60 (30 per arm) would be sufficient to detect substantial changes from our interventions [51]. As this is the first study aiming to establish an e-RPT program for AUD, this sample size calculation relies on data from face-to-face RPT and takes into account the established effectiveness of online psychotherapy compared to face-to-face psychotherapy [30].

The analysis will begin by examining the data for missing, nonsensical, and outlying variables. The data will then be analyzed on a per-protocol basis, excluding missing data, and with intention-to-treat analysis to assess the clinical effects of the program on participants who dropped out prematurely. All calculations will be done using a 2-tailed significance level of α=.05, except for when a Bonferroni correction is needed. The demographic information of program completers and program dropouts will be compared using independent sample *t* tests to identify possible differences. A 2 by 3 repeated measures ANOVA of the primary (daily drinking and relapse) and secondary (SCQ-100, Q-LES-Q-SF, RS-14, CBI, and PHQ-9) outcomes will be conducted to test for the effects of the interventions (e-RPT or face-to-face RPT) throughout the 10-week treatment (0, 5, and 10-week assessment points). Linear regression (for continuous outcomes) and binomial regression analysis (for categorical outcomes) will be used to identify variables associated with outcome measures. Additionally, OPTT will collect usage statistics (ie, number of logins per day, amount of time spent logged in) and compare them to face-to-face metrics (ie, attendance to weekly 1-hour face-to-face sessions) to determine cost and time savings between the 2 programs. All statistical analyses will be conducted using IBM SPSS Statistics for Mac (version 24; IBM Corp).

## Results

According to the literature on the efficacy of face-to-face psychotherapy compared to web-based psychotherapy in the management of AUD, we hypothesize that both e-RPT and face-to-face RPT will reduce alcohol consumption and relapse risk and improve secondary outcome measures (depressive symptomatology, self-efficacy, quality of life, and resilience) [44-47]. Recruitment and data collection are expected to end in October 2023. Data analysis will be concluded by December 2023, at which point the process of knowledge dissemination will begin, including but not limited to peer-reviewed publications, scientific presentations, grant proposals, and reports.

## Discussion

This is a protocol for an RCT that aims to establish a novel e-RPT program to improve the mental health of patients with AUD. This program will teach effective coping techniques to maintain abstinence and reduce the risk of a relapse by applying the widely validated principles of CBT and relapse prevention [12,24,31,32,50]. Supporting the structure and design of this program, this study will apply the evidence gathered by Alavi et al [30,39,40] on the efficacy of e-CBT in managing other psychiatric disorders. Thus, this study hypothesizes that this novel e-RPT program will have a similar effectiveness as face-to-face RPT at maintaining long-term abstinence and reducing the risk of experiencing a relapse.

Psychosocial interventions are one of the most commonly used methods for the treatment of mental health disorders [18]. CBT is one of the most-studied approaches for treating AUD and involves one-on-one sessions between the patient and therapist [19]. These sessions work on overcoming cravings for drinking alcohol by developing strategies that help people abstain from consuming alcohol when cravings occur. When targeted to prevent or reduce the risk of relapses after an initial alcohol reduction program, this CBT approach becomes RPT. RPT involves addressing coping skills to decrease the risk of relapse [21,50]. Additionally, both interpersonal and intrapersonal factors are addressed so that patients can develop the skills necessary to aid in preventing relapse. For instance, through the development of coping mechanisms to respond effectively to stressful situations that otherwise could have led to relapse [12,50]. However, despite these benefits, there are many obstacles to receiving face-to-face psychotherapy, such as financial issues and logistical problems.

The strengths of this program and this study include the use of asynchronous psychotherapy through predesigned web-based modules. These web-based modules have been shown to increase therapy efficiency by allowing a web-based therapist to deliver care to 3 to 4 patients at a time compared to 1 synchronous session [30,39,40]. The use of a web-based platform like OPTT allows for the automatic and reliable collection of user analytics, which can then be used to continuously improve and optimize the delivery of web-based psychotherapy programs. Given that face-to-face RPT has been previously validated in the literature, this study hypothesizes that e-RPT will be effective at maintaining abstinence, reducing the risk of relapse and depression symptomatology, and improving quality of life, resiliency, and coping behaviors [12,24,31,32,50]. Furthermore, comparing our proposed e-RPT program to a validated face-to-face therapeutic approach (RPT) strengthens the design of this RCT and the conclusions that will stem from this evidence.

Despite the strengths of this program, the web-based mode of delivery of this program may have some potential limitations. The main limitation is that participation requires a reliable internet connection and sufficient technology literacy. This limitation could introduce biases in participant demographic characteristics. Thus, this study will analyze demographic factors through multiple sample *t* tests to determine if these factors could impact treatment outcomes. Additionally, due to the delivery mode and design of the program, it is not possible to conceal client allocation. Moreover, web-based psychotherapy is often characterized by a dropout rate of up to 40% by the end of the treatment [49,50]. To account for this without compromising statistical power, this study is intentionally oversampled.

The knowledge dissemination plan for this study includes publishing the outcomes of this study in a peer-reviewed, academic journal. The research team will also attend academic meetings and conferences to present these findings and commit to continuing education in the relevant research areas. Furthermore, the dissemination and application of knowledge will be further enhanced through the collaboration of the Canadian enterprise, OPTT Inc. Additionally, by providing this service to clinicians, the care capacity for accessible, affordable, and high-quality mental health care could be more rapidly scaled across Canada.

To conclude, online psychotherapy has the potential to allow for more services to be provided at a lower cost and with increased capacity and accessibility, but research on its applications to relapse prevention is limited. Therefore, the results of this study aim to supplement the literature on the use of online psychotherapy for the management of AUD and to validate the implementation of this e-RPT program. Thus, the findings from this study can help provide the stepping stones for the large-scale implementation of programs like this one and may provide family medicine clinics, specialists, mental health professionals, and insurance companies with a new, more affordable, and accessible mental health resource.

## Appendix

Multimedia Appendix 1 Peer review report by Queen’s University Department of Psychiatry Internal Research Grant Review (Kingston, Canada).

## Abbreviations

AUD: alcohol use disorder
CBI: coping behaviors inventory
CBT: cognitive behavioral therapy
e-CBT: internet-based cognitive behavioral therapy
e-RPT: web-based relapse prevention therapy
MINI: Mini International Neuropsychiatric Interview
OPTT: Online Psychotherapy Tool
PHQ-9: Patient Health Questionnaire
PI: principal investigator
Q-LES-Q-SF: Quality of Life Enjoyment and Satisfaction Questionnaire—Short Form
RCQ: Readiness to Change Questionnaire
RPT: relapse prevention therapy
RS-14: 14-Item Resilience Scale
SCQ: Situational Confidence Questionnaire

## Data Availability

Data sharing is not applicable to this article as no data sets were generated or analyzed yet for the preparation of this protocol manuscript. Though the data sets generated during and analyzed in the randomized controlled trial will be available from the corresponding author on reasonable request.
