# Supplementary material for: Developing and Implementing a Web-Based Relapse Prevention Psychotherapy Program for Patients With Alcohol Use Disorder: Protocol for a Randomized Controlled Trial
Source: JMIR Res Protoc. 2023 Jan 25;12:e44694. doi: 10.2196/44694 (PMC9909521; doi:10.2196/44694)
Supplement: Multimedia Appendix 1 [file resprot_v12i1e44694_app1.pdf]

Queen's UNIVERSITY

Queen's University Department of Psychiatry Internal Research Grant Review

Title of Grant: Developing and implementing an Online Relapse Prevention Psychotherapy program for Patients with Alcohol Use Disorder

Primary Investigator: Dr. Megan Yang

Instructions:
Assess per the adjudication scale and list 3-5 strengths and/or weaknesses in point form in the space provided.

**Adjudication Scale**	
**4.5 – 4.9** Exceptional	**3.0-3.4** Very good however needs revision to be fundable
**4.0-4.4** Outstanding	**2.5-2.9** Needs major revision
**3.5-3.9** Excellent, may still require revision	**0.0-2.4** Seriously flawed

**Originality/Scientific Merit:**	**Adjudication Score: 4.0**

Comments: This is a fairly well-written application for a small exploratory study to assess the impact of an online cognitive-behavioural relapse prevention intervention for patients with alcohol use disorder. The work is timely and represents a logical progression in terms of the PI and co-applicants' research and career trajectories. It has the potential to produce some solid pilot data to underpin a future application for external grant funding. I appreciated the opportunity to review the application and note several strengths in my comments, as well as some potential areas for improvement, greater clarity or strengthening.

**Strengths:**	**Weaknesses:**
1) The rationale for the potential impact of the research in the context of COVID-19 is well-articulated.<br><br>2) A reasonable expected dropout rate (40%) is incorporated.<br><br>3) The efficacy of e-CBT delivered through OPTT has been previously demonstrated by members of this research team in managing other disorders including mood and anxiety; this step to evaluate the intervention in patients with AUD is logical	1) The recruitment and statistical analysis plans would benefit from inclusion of a more fulsome attention to sex-and gender sex-and gender based analysis (SGBA) plan. Will parity in male/female gender be sought in recruitment? Will non binary gender identity be assessed in terms of demographics?<br>2) Engagement with therapy will be assessed via fairly traditional but blunt metrics (i.e., number of logins per day, amount of time spent on each session). The rigour of this section would be bolstered by the inclusion of an engagement scale designed for the assessment of engagement with online interventions, such as the User Engagement Scale (UES).<br>3) It is noted that "All therapists will be research assistants hired by the principal investigator. They will all undergo training in psychotherapy and additional training from a psychiatrist on the research team before any interaction with participants". I did query the use of RAs to provide the individualized feedback sessions as this is likely to be one of the most important elements of the intervention for optimizing engagement and

	retention. On reviewing the co-investigator list, it appears that the applicant team and the RAs in particular have some baseline experience but further details of the training provided and details of ongoing supervision would have bolstered the application.<br>4) From an EDI standpoint, I would have liked to see a rationale provided for the upper age inclusion cut-off of 65 years

Impact and Research Applicant(s)

**Adjudication Scale (Research impact and Research Applicant(s))**	
**4.5–4.9** Extremely significant impact appropriate team	**3.0-3.4** Moderate impact and/or poor team
**4.0-4.4** Very significant impact ,appropriate team	**2.5-2.9** Limited impact and/or poor team
**3.5-3.9** Significant impact, appropriate team	**0.0-2.4** Negligible impact and/or poor team

**Potential Impact and Team:**	**Adjudication Score: 4.0**
Comments: Potential impact is there in the project, but would be stronger with a little more attention to rigour in terms of description of project methods. The team composition is strong.	
**Strengths:**	**Weaknesses:**

1) An important area for research focus that's timely given the data we have on alcohol use rates during COVID-19 in Canada and current attention to ehealth and mhealth care delivery.<br>2) Dr. Yang's research focuses on the effectiveness of e-CBT in the treatment of mood and anxiety disorders; this award seems like a logical stepping stone in terms of expansion of her research focus. Co-applicant Dr. Alavi has more than 12 years of research experience in delivering online psychotherapy with a focus on e-CBT and e-DBT.<br>3) The team contains a good mix of clinician scientists, co-applicants with statistical expertise and trainees; the research environment should provide ample training and capacity building opportunities.	1) It is noted that "All therapists will be research assistants hired by the principal investigator. They will all undergo training in psychotherapy and additional training from a psychiatrist on the research team before any interaction with participants". I did query the use of RAs to provide the individualized feedback sessions as this is likely to be one of the most important elements of the intervention for optimizing engagement and retention. On reviewing the co-investigator list, it appears that the applicant team and the RAs in particular have some baseline experience but further details of the training provided and details of ongoing supervision would have bolstered the application.<br>2) I would have liked to have seen a plan for the inclusion of lived experience expertise in the application and some attention to patient-orientated research methods. How will "engaging with end-users" as metioned in the budget occur?<br>3) A forward-thinking section on potential limitations of the research (for example, subjective reporting of alcohol consumption) and potential mitigation strategies would have been an asset in terms of determining potential impact.<br>4) Four hypotheses are presented with a number of outcome assessments nested under some of the hypotheses. A primary outcome hypothesis should have been articulated in order to maxmise the potential impact of project outputs.

Overall Score (based on CIHR scale below)

Descriptor	Score	**Outcome**
Outstanding	4.5 – 4.9	**May Be Funded**
Excellent	4.0 – 4.4	
Very good	3.5 – 3.9	
Acceptable, but low priority	3.0 – 3.4	**May or May Not be Fundable**
Needs revision	2.5 – 2.9	
Needs major revision	2.0 – 2.4	
Seriously flawed	1.0 – 1.9	
Rejected	0.0-0.9	

**Overall Adjudication Score: 4.0**
Comments:
